# Biodegradable Colorimetric Indicative Films Based on Kurugua (*Sicana odorifera*) Peel Powder

**DOI:** 10.3390/polym17091167

**Published:** 2025-04-25

**Authors:** Orlando Duarte, Germán Ayala Valencia, Omayra B. Ferreiro, Shirley Duarte

**Affiliations:** 1 Faculty of Chemistry, National University of Asunción, San Lorenzo 1055, Paraguay; orlyduart@gmail.com; 2 Department of Chemical and Food Engineering, Federal University of Santa Catarina, Florianópolis 88040900, SC, Brazil; g.ayala.valencia@ufsc.br; 3 Bio and Materials Laboratory, Polytechnic School, University of Asunción, San Lorenzo 2111, Paraguay

**Keywords:** colorimetric indicator films, kurugua peel, anthocyanins, color change of the films

## Abstract

Colorimetric films are helpful as indicators of the freshness of foods, changing color as their pH varies as they undergo decomposition reactions. Anthocyanins are an important group of bioactive compounds whose color varies depending on the pH of the medium, which is why they are used in this type of film. This work evaluated colorimetric indicator films based on the cassava starch and anthocyanins present in kurugua (*Sicana odorifera*) peel powder. The total anthocyanin content in the kurugua peel powder was quantified (12.13 ± 0.48 mgC3G/L of extract). Films were prepared by casting, based on starch without (PC) and with the addition of kurugua shell powder in low (PB) and high (PA) proportions. Adding kurugua peel powder affected the properties of the films, such as their thickness (0.08 to 0.13 mm), solubility (15 to 18%), humidity (21 to 23%), water contact angle (31 to 61°), density (0.17 to 0.33 g/cm^3^), and opacity (2.73 to 7.74 A600 nm/mm). Raman spectra showed characteristic peaks for starch and anthocyanins. Finally, the color change capacity of the colorimetric indicator films (PA and PB) was demonstrated, changing to green and yellow colorations at high pH values, as well as during their application in monitoring the freshness of chicken meat; thus, their suitability for use as active and intelligent indicators in the food industry was confirmed.

## 1. Introduction

The food industry is constantly searching for viable and environmentally friendly alternatives for food preservation, such as the preparation of biodegradable films, which also contributes to the environmental problems generated by conventional plastics. Starch is a polysaccharide widely used as a biopolymer for the manufacture of biodegradable packaging, which has been used for several years due to its gelatinization characteristics and properties, low cost, good degradable properties, biocompatibility, and abundance [1,2].

Cassava is a root whose global production reached 330 million tons in 2022; it is the second starch source worldwide, only after corn, and is mainly used as native starch [3,4]. Cassava starch has a yield of around 80% of the dried weight of cassava root and a high proportion of amylose compared to other sources, which gives it considerable industrial importance [5]. This characteristic allows it to form strong, opaque gels with a capacity for syneresis. In the same way, the transition of industries towards a circular economy and sustainability approach is imminent, and taking advantage of the waste generated within the food industry is an opportunity for this transition.

According to the food waste index published by the United Nations Environment Program (UNEP), 931 million tons of food are wasted worldwide yearly, equivalent to 17% of the total food available to consumers [6], among which are the peels or skins of some fruits. Kurugua (*Sicana odorifera*) is a native fruit from Paraguay. It has the potential to be industrialized due to its nutritional and medicinal properties, being used in some countries to treat liver diseases, the respiratory system, and throat issues, and even to combat venereal disease disorders and uterine bleeding [7], in addition to its antioxidant properties, due to it being rich in anthocyanins and polyphenol compounds [8].

Mereles et al. [8] characterized the kurugua peel of ripe fruits growing in Paraguay. The authors reported a moisture content of 8.84 g/100 g, ash of 3.95 g/100 g, and total lipids of 10.58 g/100 g, while its total monomeric anthocyanin was 19.7 mg cyanidin 3-glucoside/g, its total phenol compounds were 100 mg GAE/100 g, and it had an antioxidant capacity ABTS of 0.201 µM TEAC/g. Anthocyanins are non-nitrogenous plant compounds that belong to the flavonoid family and are widely distributed in nature; for example, they are present in the peel of certain fruits, such as pears and apples, or in the fleshy part, such as strawberries and plums [9].

Anthocyanins act as pH indicators due to a deficiency in the flavin nucleus. Generally, they exhibit red coloration at acidic pH due to a stable oxonium structure of colored flavin cation. With increasing pH, deprotonation of the flavin cation is promoted: at neutral pH, they show pink coloration, and at basic pH, they show blue or purple coloration due to continuous deprotonation where quinoidal forms predominate, and the bathochromic effect is considerable [2,9,10]. Anthocyanin extract at pH above 7 gradually changes the coloration from purple to blue, due to the formation of quinoidal anhydrobase, and finally, to green and yellow because of anthocyanin degradation in alkaline media [11]. Wahyuningsih et al. [12] graphically illustratees the structural changes that occur in the basic anthocyanin framework as a function of pH.

This characteristic is ideal for visually observing the deterioration of certain foods. Therefore, the incorporation of anthocyanins from the peel of certain fruits into biodegradable films to convert them into intelligent colorimetric indicator films has been a matter of interest for several authors [12,13,14,15]. Intelligent packaging can be incorporated into food packaging structures as labels inside a package to monitor changes in the acidic and basic components of a food product. These packages allow the consumer to check and monitor in real-time the quality of foods such as bread or dairy [16,17] and control the freshness or spoilage of meat products [11,13,18,19]. Different colorants have been used in intelligent films, including anthocyanins, betalains [20], and carotenoids [21]. All these colorants show a color dependence on the pH of the medium. One of the main advantages of anthocyanins is their solubility in water, which allows the preparation of films using natural biopolymers known to be hydrophilic. In addition, in this work, the kurugua peel was used without further processing, which highlights the direct use of the residue from the processing of this fruit [22]. According to the FAO, world meat consumption is dominated by chicken meat, thanks to its accessibility, low-fat content, and almost no religious or cultural restrictions. In addition, it contributes to human nutrition by providing high-quality protein and desirable fatty acids. It is also rich in phosphorus, other minerals, and B vitamins [23,24]. The pH value for fresh chicken meat ranges from 5.96 to 6.18 [25]. This parameter is important in the food industry because it allows us to control the stability of the product. This work focuses on developing and characterizing intelligent color indicator films for monitoring the freshness of chicken meat. These films were made from cassava starch and anthocyanins extracted from kurugua peel powder.

## 2. Materials and Methods

### 2.1. Materials

Biodegradable films were prepared using a combination of starch and kurugua powder (*Sicana odorífera*). The cassava starch (*Manihot esculenta*) was purchased from the CODIPSA industry (Caaguazú, Paraguay). Kurugua fruits were purchased from the local market in Paraguarí, Paraguay. Distilled water and hydrochloric acid (37 wt%) were used as solvents. Glycerol P.A. was used as a plasticizer in the preparation of the films.

### 2.2. Quantification of Anthocyanins from Kurugua Peels

The kurugua fruits were washed using distilled water and dried with absorbent paper. The peels of the kurugua were manually separated and then dried in an oven at 40 °C for 24 h and finally milled in a stainless steel knife mill (IKA A11 Basic). Ground peels were sieved (80 mesh = 180 μm), packaged inside polyethylene bags, and stored at 4 °C under the absence of light until analysis.

The total monomeric anthocyanin (TMA) content in the kurugua peel powder was quantified from the extract obtained as described by Merz et al. and Capello et. al. [12,13], with modifications. A solid-liquid extraction was performed using approximately 0.5 g of dried kurugua peel powder in 100 mL of an acidified aqueous solution (100:1 *v*/*v*, H_2_O: HCl 37 wt%), with continuous stirring (100 rpm) in the absence of light for 60 min at 35 °C. The extract was separated by centrifugation (2790 g). Aliquots (0.5 mL) were dissolved in 4.5 mL of 0.025 M potassium chloride buffer (pH = 1) and 4.5 mL of 0.4 M sodium acetate buffer (pH = 4.5) in separate test tubes. The absorbance of the extracts was sampled and measured at 522 nm (A522 nm) and 699 nm (A699 nm).

The total anthocyanin content was quantified using the differential pH method and spectrophotometric analysis, according to Giusti and Wrolstad [26]. The absorbance of the sample and the concentration of total monomeric anthocyanins (*TMA*) expressed in mg cyanidin-3-glucoside (C3G)/L of extract were calculated by the following equations, respectively:

(1) $$A=(A_{\lambda 522}-A_{\lambda 699}{)}_{pH1}-(A_{\lambda 522}-A_{\lambda 699}{)}_{pH4.5}$$

(2) $$TMA=\frac{A\cdot MM\cdot FD\cdot 1000}{\varepsilon \cdot L}$$

where A represents the calculated absorbance from Equation (1); *MM* is the molar mass of the predominant anthocyanin in the sample (cyanidin-3-glucoside: 449.2 g/mol); *FD* is the dilution factor; ε represents the molar absorptivity, and *L* is the optical path length equal to 1 cm.

### 2.3. Preparation of Color Indicator Films

The films were prepared according to the methodology described by Talja [27], with modifications. Cassava starch (4 g/100 mL), glycerol (1.2 g/100 mL), two proportions of kurugua peel powder (0.25 and 0.5 g/100 mL), and distilled water were used to prepare the filmogenic solutions. Solution A, consisting of cassava starch, glycerol, and 70 mL of distilled water, was subjected to continuous stirring in a thermostatic bath at 70 °C for 30 min; at the same time, Solution B was prepared with the help of an electric mill by adding the required amount of kurugua peel powder with 30 mL of distilled water. The solution in the thermostatic bath was cooled to 40 °C and both solutions were mixed under continuous stirring for 5 min. Then, the resulting solution was cast in a 14 × 19 × 0.03 cm^3^ acrylic mold and dried under a laminar flow hood for 48 h at room temperature. A control film was prepared without the addition of kurugua peel powder.

### 2.4. Characterization of Films

#### 2.4.1. Morphology, Film Thickness, Moisture Content (MC) and Density

The air-contact surface of the control and colorimetric indicator films was analyzed using a Zeiss Evo15 (Oberkochen, Germany) scanning electron microscope (SEM) at an accelerating voltage of 5 kV. For analysis, the films were fixed on aluminum stubs with carbon tape and then coated with a thin layer of gold. The samples were coated using conductive material (Au/C) coating equipment, Quorum, Model Q150R ES Plus (East Sussex, UK). Micrographs were taken at random sample points at 150× magnification following the work available in the literature [12,13,28].

Film thickness was measured using a micrometer (Mitutoyo, Kawasaki City, Japan) with a precision of 0.001 mm. Measurements were taken at 10 different sample points, and the mean and standard deviation were calculated afterward. MC was analyzed using a moisture balance (MOC-63U, Tokyo, Japan) by drying films at 105 °C. MC values were expressed as a percentage with a precision of 0.001 g.

Film density (ρ) was determined using pieces of film (6 × 4 cm^2^ area) with known initial weight ($w_{i}$). The final weight of the films ($w_{f}$) was determined after oven drying at 105 °C for 24 h [19]. The value of *ρ* was calculated using Equation (3)

(3) $$\rho =\frac{W}{V}=\frac{W_{i}-W_{f}}{S\cdot e}$$

where *S* and *e* are film surface and thickness, respectively.

#### 2.4.2. Solubility in Water

Film solubility in water was determined using three samples of approximately 2 cm diameter (known initial dry weight). Samples were immersed in distilled water (50 mL) and placed in a shaker (Solab, SL-222, São Paulo, Brazil) under slight stirring (100 rpm) for 24 h at 25 °C. The material was filtered and then dried in an oven at 105 °C for 24 h for determination of the final dry weight. Solubility (%) was then calculated as dry weight difference.

#### 2.4.3. Optical Properties

Film transparency was determined using a UV-Vis spectrophotometer (HITACHI U-1900, Tokyo, Japan) with a wavenumber at 600 nm. Film samples were fixed in the cuvette in such a way that the light beam passed through the film. Opacity was determined as the film absorbance at 600 nm divided by film thickness and expressed as Abs 600 nm/mm [19].

Film color characterization was carried out using a colorimeter (Delta Vista 450G, Delta Color, São Paulo, Brazil) with the CIELab coordinates represented by L* (represents lightness index), a* (represents the tonalities from green to red color), and b* (represents the tonalities from blue to yellow color). The color difference was calculated using Equation (4)

(4) $$\Delta E*=\sqrt{(\Delta L*{)}^{2}+(\Delta a*{)}^{2}+(\Delta b*{)}^{2}}$$

where: $\Delta L*=L_{standard}^{*}-L_{film}^{*}$; $\Delta L*=a_{standard}^{*}-a_{film}^{*}$; and $\Delta L*=b_{standard}^{*}-b_{film}^{*}$.

In this study, the color parameters of the control films were considered as the standard [13,14].

#### 2.4.4. Surface Hydrophilicity

The surface hydrophilicity of films was determined by means of water contact angle (WCA) analyses according to the ASTM D7334 standard, using an optical tensiometer (RaméHart 250, Succasunna, NJ, USA) [28]. The films were attached to the equipment and a drop of distilled water of approximately 5 μL was released over the air surface of the films using a micropipette. The angle formed between the surface of the film and the tangent to the drop was calculated using DROPimage Advanced software (RaméHart, USA). The water contact angle measurements were made by analyzing the shape of each sessile drop after it had been placed over the samples for 10 s. A total of three measurements were taken per film.

#### 2.4.5. Chemical Bonds and Crystallinity

Raman spectra were obtained at room temperature using an Anton Paar Spectroscopy Suite Raman software (Cora 5200, Edison, NJ, USA), coupled with a red solid-state laser as the excitation source (λ = 785 nm), and operating with a laser power of 400 mW. Raman spectra were obtained in the region between 100 and 2300 cm^−1^.

### 2.5. Application of Colorimetric Indicator Films for Monitoring the Freshness of Chicken Meat

The efficiency of the colorimetric indicator films was evaluated by the color change by performing monitoring of the freshness of chicken meat according to Merz et al. [12]. Pieces (6 × 4 cm) of fresh meat were placed inside Petri dishes and sealed using a Petri dish cover containing the colorimetric indicator film (2 × 2 cm) placed on top. The Petri boxes were kept refrigerated (4 °C) and in an artificial climate incubator (30 °C) [12,13].

The color change of the film was monitored both visually and by colorimetry. The pH of the meat was measured using a digital pH meter (Testo 205, São Paulo, Brazil) for semisolid samples at six different spot positions on each piece of chicken meat for 0, 24, 48, 48, 72, and 96 h, according to Qin et al. [29].

### 2.6. Statistical Analysis

For the statistical analysis of the data, first, the characteristics of the three types of films obtained according to the kurugua powder content (independent variable) were analyzed as follows:

- Group 1 corresponds to the control film (PC), obtained without adding the kurugua powder;
- Group 2 corresponds to Film B (PB), obtained by adding 0.25 g of kurugua powder;
- Group 3 corresponds to Film A (PA), obtained by adding 0.5 g of kurugua powder.

The measurements of the dependent variables were carried out minimally in sextuplicate for each determination, and the statistical analysis was performed to determine the type of behavior presented and observe the existence or absence of significant differences between the study groups as follows:

i. The Ryan–Joiner normality test was performed, where if *p* > 0.01, they comply with a normal distribution.
ii. The test for homogeneity of variances (Levene or other) was performed, where if *p* > 0.01, the variances are equal.
iii. If they complied with both hypotheses, it was confirmed that the data presented a parametric distribution, and point iv was performed. If they presented a non-parametric distribution, point v was considered.
iv. ANOVA test. Where if *p* > 0.01, all population means are similar. If *p* < 0.01, there is a significant difference between the population means and Tukey’s test comparing which means are equal.
v. Kruskall–Wallis test to determine if the median is equal among the three groups, followed by a Dunn comparison test to determine which groups produce statistically significant effects.

According to the results obtained, the film with the best characteristics for monitoring the freshness of chicken meat was chosen. For this determination, a longitudinal analysis was carried out at different temperatures. Other population groups corresponding to these storage temperatures or independent variables were available:

- Group 4 corresponds to a refrigerated storage temperature equal to 4 °C.
- Group 5 corresponds to a storage temperature in an artificial incubator equal to 30 °C.

The chicken meat’s pH and the color parameters (L*, a*, b*) at each temperature were measured in sextuplicate. Following points i to v, statistical analysis was performed. Data processing was carried out using the statistical program Minitab 18 Statistical Software (Minitab, LLC, State College, PA, USA). The OriginPro 8 (OriginLab Corporation, Northampton, MA, USA) program was used for the results graphs.

## 3. Results and Discussions

### 3.1. Anthocyanins Content in Kurugua Peel Powder

#### Total Monomeric Anthocyanin (TMA)

Table 1 shows the absorbance values measured at different wavelengths (522 nm and 699 nm) and pH (1 and 4.5), along with the TMA content, calculated by Equation (1).

The calculated average TMA value was 12.13 ± 0.48 mg/L for the dry kurugua peel powder. This value is higher than that reported by Capello et al. [13] for sweet potato (*Ipomoea batatas* L.) (6.93 ± 1.25 mg/L) and lower than that obtained for yvapurú (*Plinia cauliflora*) (54.61 ± 1.00 mg/L). These results demonstrate the potential of kurugua peel powder (*Sicana odorifera*) as a valuable source of anthocyanins for the development of colorimetric indicator films.

### 3.2. Physicochemical Characterization of Films

#### 3.2.1. Chemical Structure

**Figure 1:**
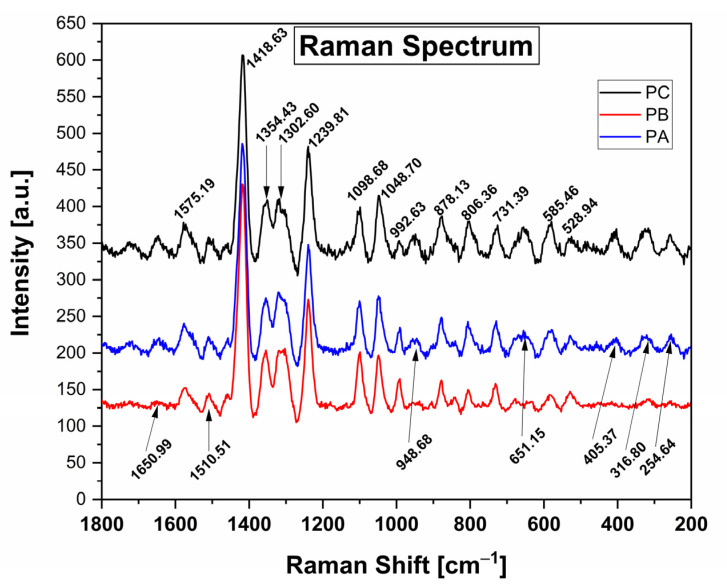
Raman spectra of the control films (PC) and films with added Kurugua peel powder; low and high (PB and PA, respectively).

Figure 1 presents the Raman spectra of the control film (PC) and films containing low (PB) and high (PA) quantities of kurugua peel powder. The spectra exhibit signals at wavenumbers between 528.94 cm^−1^, as well as bands at 1239.81 cm^−1^ and 1418.63 cm^−1^. Analysis of the spectra reveals that all films show peaks at similar wavenumbers, indicating consistent peak positions. However, slight variations in intensity are observed for the PB and PA films compared to the control film (PC).

Among the most significant signals, compared with those reported by Cael et al. and others [30,31,32,33] for films based on cassava starch, the spectrum reveals prominent peaks at 1418.63 cm^−1^ and 1239.81 cm^−1^, corresponding to C-H bending vibrations and CH_2_ deformation, respectively. Additionally, lower intensity peaks at 1354.43 cm^−1^ are attributed to C-O-H bending vibrations, while those at 1302.60 cm^−1^ are associated with C-H bending vibrations. The peaks observed at 1098.68 cm^−1^ and 1048.70 cm^−1^ correspond to C-O-H bond bending. Further, the peaks at 992.63 cm^−1^, 878.13 cm^−1^, and 806.36 cm^−1^ are indicative of an alpha configuration for C-1-H bending vibrations. Lastly, the peak at 731.39 cm^−1^ is associated with C-C bending vibrations.

In the films containing kurugua peel powder, additional characteristic peaks corresponding to the bonds present in anthocyanins are observed. The peak at 1650.99 cm^−1^ is associated with the stretching of the benzene ring and the hydrogen substitution of OH groups at positions 3 and 7 [33]. The peaks at 651.15 cm^−1^, 405.37 cm^−1^, 316.80 cm^−1^, and 254.84 cm^−1^ are attributed to glucose vibrations [31]. The peak at 948.68 cm^−1^ corresponds to C-C vibrations, while the peak at 1510.51 cm^−1^ is associated with CH_2_ vibrations [34].

#### 3.2.2. Morphology, Thickness, and Density

**Figure 2:**
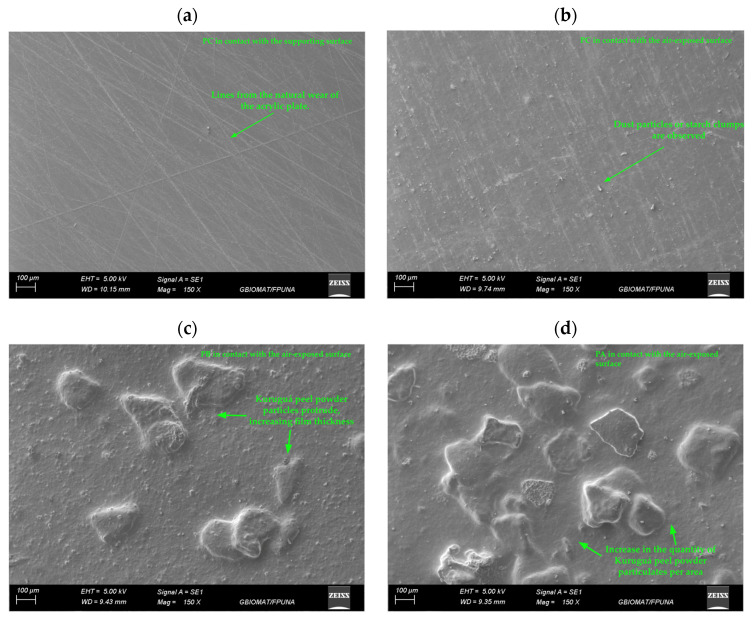
Micrographs of films: (**a**) control film (PC) in contact with the supporting surface and (**b**) in contact with the air-exposed surface; (**c**) film with low kurugua powder content (PB) in contact with the air-exposed surface; (**d**) film with high kurugua powder content (PA) in contact with the air-exposed surface.

Figure 2 shows scanning electron microscopy (SEM) images of cassava starch-based films, with and without the incorporation of kurugua peel powder.

The control films (PC) exhibited a morphology characterized by a uniform, compact, and non-porous matrix on the surface in contact with the mold (Figure 2a). Additionally, noticeable lines were observed, which were attributed to the natural wear of the acrylic plate used as a mold during the fabrication process.

In contrast, the air-exposed surface of PC film (Figure 2b) displayed small dust particles, likely deposited by gravity from the external environment. Alternatively, these particles might result from the formation of clumps during the initial dissolution of starch in cold water before heating.

The PA and PB films, incorporating kurugua peel powder, exhibited similar characteristics to the control film on the surface in contact with the mold, including lines associated with mold wear. However, the air-exposed surface (Figure 2c,d) showed significant alterations in morphology, with rough and heterogeneous textures attributed to the incomplete solubilization of the powder particles. Notably, the PA film exhibited a higher degree of particle aggregation compared to the PB film, directly correlating with the higher kurugua peel powder content in its formulation.

Similarly, Luchese et al. [14] developed starch-based films using yvapurú flour as a source of anthocyanins and observed rough surfaces; however, these did not affect the color variation of the films as a function of pH.

The thickness of the film was significantly influenced by the incorporation of kurugua peel powder. A notable increase in thickness was observed in the colorimetric indicator films compared to the PC. This result can be attributed to the higher amount of solids introduced into the polymer matrix with the addition of kurugua peel powder. However, no statistically significant differences were identified in thickness with increasing powder content among the colorimetric indicator films PA and PB (Table 2). These findings align with those previously reported by Gaviria et al. and Nogueira et al. [19,35].

Regarding the film density (Table 2), the values decreased as the concentration of kurugua peel powder increased. This phenomenon may be attributed to the anthocyanins or other substrates present in the powder, which could increase molecular spacing within the amylose and amylopectin chains of the film matrix. Such changes alter the compact structure of the film, leading to variations in density. Similar results were reported by Leiva Ramos et al. and others, who observed higher or lower densities in their developed films compared to their control films [36,37].

#### 3.2.3. Moisture Content (MC), Solubility in Water (SW), and Water Contact Angle (WCA)

The moisture content of all the films ranged from between 21% and 23%, with no significant effect observed from the addition of kurugua peel powder (Table 2). Similarly, Merz et al. [9] reported comparable moisture content values in their films developed using chitosan, polyvinyl alcohol, and anthocyanins extracted from jambolan.

The water solubility percentage showed a significant increase in the PA film, which contained a higher concentration of kurugua peel powder, compared to the control film (PC) and the film with a lower powder content (PB), between which no significant differences were observed (Table 2). Similarly, Nogueira et al. [35] reported a significant increase in solubility values with the progressive addition of blackberry powder in their films compared to the control film. Based on the results of both studies, it can be inferred that the low solubility observed in films containing kurugua peel powder could be attributed to the low hydrophilicity of the powder particles, which are incorporated into the colorimetric indicator film, as previously illustrated in Figure 2. It has been reported that a low level of solubility of films containing anthocyanins or other bioactive compounds may allow for the release of these compounds, which could be beneficial in the preservation of foods that are stored with these films [38].

The water contact angle (WCA) values obtained for the three films showed significant differences (Table 2). However, all the values were below 90° [39,40], indicating that the films can be classified as wettable or hydrophilic surfaces, making them unsuitable as coatings for food products with high water content.

**Figure 3:**
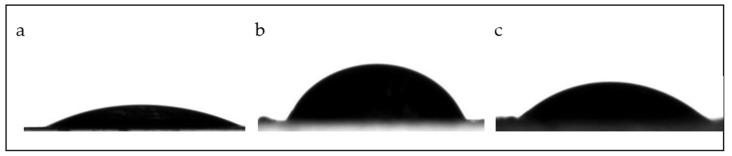
Sessile drop images applied to the air-exposed surfaces of the studied films: (**a**) PC film, (**b**) PB film, and (**c**) PA film.

Additionally, the WCA measured for the film with the highest content of kurugua peel powder (PA) was lower compared to the film with a lower powder content (PB) (Figure 3). This reduction can be attributed to the greater surface roughness observed in the PA film, which enhances its surface hydrophilicity [41].

#### 3.2.4. Thermal Properties

**Figure 4:**
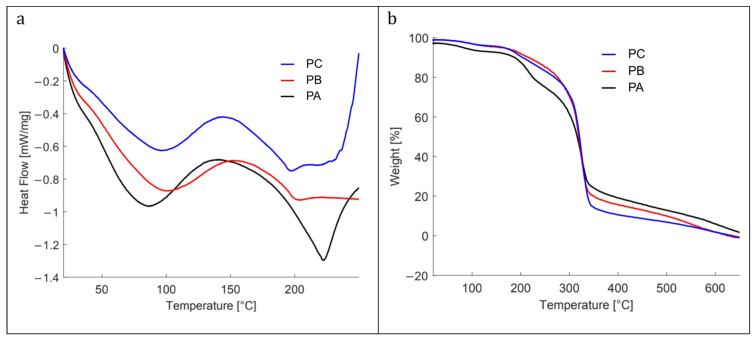
(**a**) Differential scanning calorimetry, and (**b**) differential thermogravimetric analysis of the studied films: control film (PC), with low (PB) and high (PA) content of kurugua peel powder.

Figure 4 shows the thermograms obtained from the DSC and TGA analyses of the colorimetric indicator films and the control film.

The DSC heat flow curves for all materials revealed characteristic endothermic peaks for starch-based films within 40 °C to 125 °C, corresponding to the melting of starch crystals formed during retrogradation [42]. For the PA film, which contained the highest concentration of kurugua powder, the endothermic peak shifted slightly to the left, indicating a lower melting temperature. This shift suggests that the addition of kurugua powder limited starch recrystallization by interacting with the polymer chains and disrupting their alignment.

Additional endothermic peaks were observed at higher temperatures, notably between 175 °C and 230 °C, and were primarily associated with the decomposition of glycerol and starch polymers, as described by Liu et al. [43]. The TGA results further confirmed that all the materials analyzed via DSC remained thermally stable up to approximately 200 °C.

Figure 4b illustrates the thermal decomposition of the three films in three main stages. The first stage (30–175 °C) corresponds to the evaporation of water and/or volatile substances. At 100 °C, the weight loss is 3% for PC and PB films, while PA reaches 6%. At 175 °C, the weight loss increases to 6% for PC and PB, and to 9% for PA.

The second stage (175–275 °C) is associated with the decomposition of the glycerol-rich phase, which also contains starch, and the degradation of low-molecular-weight components from kurugua powder. During this stage, the PC and PB films exhibit a weight loss of approximately 20%, whereas PA loses 30%.

The third stage (275–350 °C) corresponds to the degradation of starch components. At 325 °C, the three films (PC, PB, and PA) converge at a weight loss of approximately 60%. Finally, at 350 °C, the total weight losses are 86% for PC, 80% for PB, and 76% for PA. Although slightly different, these results align with those reported in the literature for starch- and glycerol-based films [42,44,45].

#### 3.2.5. Optical Properties

**Figure 5:**
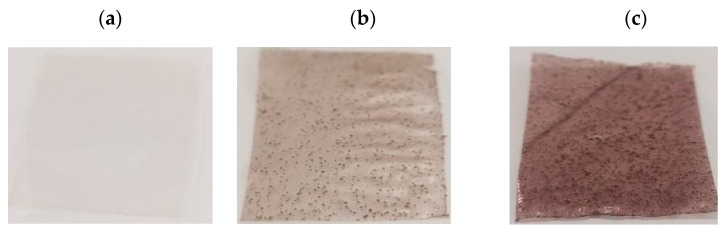
The visual appearance of the films: (**a**) control film (PC), (**b**) film with a low concentration of kurugua peel powder (PB), (**c**) film with a high concentration of kurugua peel powder (PA).

Films produced through the casting process using cassava starch, glycerol, and kurugua peel powder formed continuous materials that were easily detached from the acrylic molds used during their fabrication. The control film (PC) exhibited a gray coloration typical of starch-based materials (Figure 5a) [19]. The addition of kurugua peel powder imparted reddish/brown tones, with intensity increasing according to the powder concentration (Figure 5b,c), likely due to the presence of anthocyanins in the peel powder.

The color parameters for PC, PB, and PA (Table 3) showed a decrease in L* values with higher concentrations of kurugua powder, indicating reduced luminosity. Conversely, the a* and b* values increased, suggesting a shift toward reddish and yellowish hues [46]. The PA film exhibited more intense coloration than PB, which is consistent with its higher powder content. Calculated ΔE* values indicate that all films containing the powder displayed colorimetric differences perceptible to the human eye (ΔE* > 3) [12,14,18].

Opacity (Op) was another optical property influenced by the presence of the kurugua peel powder, with Op values increasing as the powder concentration rose (Table 3). In this study, PA and PB films, characterized by high L*, a*, and Op values, demonstrate potential for applications aimed at reducing discoloration, nutrient loss, and undesirable flavors in foods exposed to visible and ultraviolet light [12,19].

**Figure 6:**
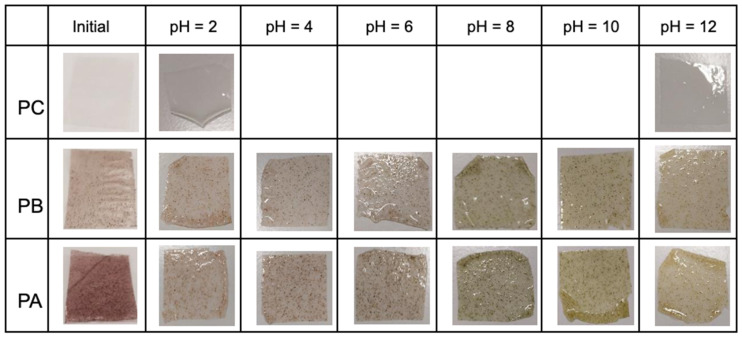
Visual appearance of control and colorimetric indicator films prior to contact with buffer solutions at different pH values (ranging from 2 to 12).

On the other hand, the color change of the films was evaluated within the pH range of 2 to 12. Figure 6 qualitatively shows the color change after exposing the films to buffer solutions for 30 min. Both films containing kurugua peel powder exhibited a rapid color variation, occurring approximately within 2 to 3 s after exposure to the solutions.

Table 4 presents the color change data. The colorimetric indicator films PA and PB show high L* values, indicating significant brightness. Regarding the a* values corresponding to chroma, an inverse relationship with pH was observed: as the pH increased, the a* values decreased. This indicates that the red hue is more pronounced at lower pH values, while green dominates at higher pH levels. For the b* chroma values, positive values were observed that increased with rising pH, indicating a dominant yellow hue at higher pH levels.

For both films studied, visually perceptible changes were detected by the human eye, and corroborated by calculated ΔE* values > 3 for each pH level, with the sole exception of PB exposed to the buffer solution at pH = 4, which exhibited a ΔE* value < 3 [12,14,18].

The observed color changes are closely linked to molecular alterations in the anthocyanin structure as a function of pH variation. The flavylium cation predominates under acidic conditions (pH 2 to 4), imparting reddish hues. At intermediate pH values (4 to 6), the molecule undergoes hydration, leading to the formation of the pseudobase carbinol, resulting in a colorless indicator. As the pH increases (8 to 10), a quinoidal structure predominates, producing a green hue. At higher pH values (10 to 12), the central ring opens, forming the chalcone structure, which imparts a yellow coloration [18,19,47].

#### 3.2.6. Application of the Colorimetric Indicator Films for Monitoring Chicken Meat Freshness

Films containing 0.5 g/100 mL of kurugua peel powder, corresponding to the PA film, were applied as they demonstrated superior color change properties during evaluation in buffer solutions.

**Figure 7:**
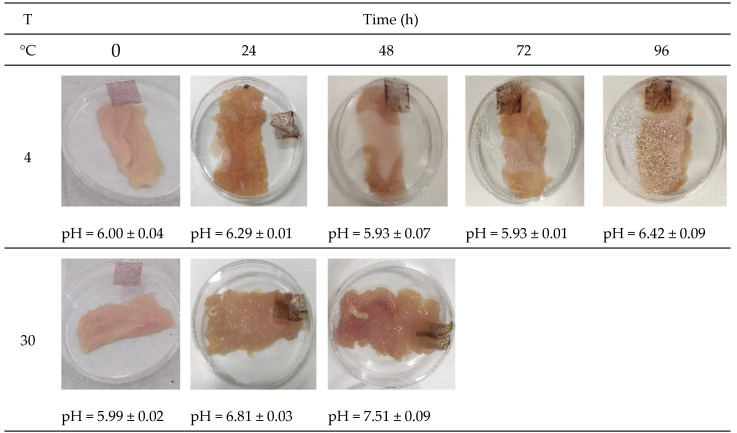
Color change of cassava starch films with kurugua peel (0.5 g/100 mL), used for monitoring the chicken meat freshness at different temperatures.

The visual appearance of the chicken meat was analyzed over 96 h at two different storage temperatures to simulate real storage conditions, using the PA film as a colorimetric indicator (Figure 7). At the start of storage (0 h), chicken meat stored at both 4 °C and 30 °C exhibited pH values around 6.00, characteristic of fresh chicken meat.

During storage at 4 °C, pH values fluctuated within the range of 5.93 to 6.42. Regarding the measured color parameters (Table 5), a significant increase in L* values was observed, indicating enhanced brightness of the colorimetric indicator film. The a* values decreased over storage time, reflecting an increase in the intensity of reddish hues. Finally, b* values progressively increased, indicating a shift toward green-yellow tones.

Similarly, the freshness of chicken meat stored at 30 °C was monitored. After the first 24 h, the pH increased from 5.99 to 6.81, and after another 24 h, it reached values close to 7.51. Samples exhibited leachates and unacceptable odors characteristic of spoiled food, limiting the colorimetric film study under these conditions to 48 h. For these samples, L* values significantly increased, indicating higher luminosity. The a* values showed a more pronounced decrease compared to samples stored at 4 °C, while the b* values significantly increased, indicating predominantly yellow tones (Table 5).

Similar results have been reported in previous studies. Merz et al. [12] observed comparable trends during shrimp freshness monitoring, while Gaviria et al. [19] and Capello et al. [18] documented analogous findings for beef freshness using developed colorimetric films. This color modification is due to microbial damage at refrigeration or higher temperatures when proteins are highly susceptible to bacteria growth, producing several volatile nitrogenous compounds such as ammonia and amines, which have alkaline pH and can induce the formation of carbinol bases in anthocyanins, resulting in color changes in the films.

It is worth mentioning that ΔE values higher than 5.0 can be detected by the human eye [48]. Therefore, based on the observed color changes in the PA film during chicken meat storage, its application as a colorimetric indicator component in food packaging is suitable. This approach has the potential to provide visual information about the freshness of chicken meat, facilitating its monitoring during storage.

## 4. Conclusions

In this study, biodegradable colorimetric indicator films were successfully developed using kurugua peel powder at two concentrations (0.25 and 0.5 g/100 mL of film-forming solution). The anthocyanin content of the kurugua peel powder was quantified at 12.13 ± 0.48 mg/L, confirming its potential as a viable source of anthocyanins for fabricating colorimetric indicator films.

The incorporation of kurugua peel powder significantly influenced the films’ physicochemical properties. A notable increase in film thickness from 0.08 ± 0.01 mm to 0.13 ± 0.01 mm (over 60%) was observed, while the water solubility, water contact angle, and film density were also affected. However, the moisture content remained consistent throughout all the films. The powder addition resulted in enhanced opacity and measurable color variations proportional to the powder concentration.

Structural characterization using Raman spectroscopy revealed similar spectra for all the films, identifying the functional groups present in the material. Thermal stability was confirmed through TGA analysis, supporting the films’ potential robustness in practical applications. Importantly, the films demonstrated distinct and visually perceptible color changes when exposed to buffer solutions of varying pH, with these changes also quantitatively validated.

The application of the kurugua-based films as freshness indicators was further assessed by monitoring the pH variations and visual color changes during chicken meat storage. The films effectively reflected changes in meat freshness, enabling both qualitative and quantitative evaluations of the color variations.

Based on the findings of this research, the developed films exhibit significant potential as complementary materials for primary food packaging, serving as reliable freshness indicators. This innovation could provide consumers with valuable, real-time information about the freshness of food products, enhancing food quality and safety monitoring.

## Figures and Tables

**Table 1:** Measured absorbance values and calculated TMA.

Measured Absorbance *				Calculated Absorbance **	TMA *** (mg/L)
pH = 1		pH = 4.5			
522 nm	699 nm	522 nm	699 nm		
0.1180	0.0030	0.0440	0.0050	0.0760	12.69
0.1150	0.0030	0.0450	0.0040	0.0710	11.86
0.1180	0.0050	0.0470	0.0050	0.0710	11.86
mean				0.727	12.13
standard deviation				0.0029	0.48

* Measurement performed in triplicate. ** Calculated by Equation (2) *** Total monomeric anthocyanin.

**Table 2:** Thickness, density, moisture content (MC), solubility in water (SW), and water contact angle (WCA) of the films.

Film	Thickness (mm)	Density (g/cm^3^)	MC (%)	SW (%)	WCA (°)
PC	0.08 ± 0.01 ^B^	0.326 ± 0.060 ^A^	21.36 ± 4.08 ^A^	15.57 ± 0.98 ^B^	31.63 ± 0.81 ^C^
PB	0.12 ± 0.01 ^A^	0.199 ± 0.016 ^B^	23.01 ± 2.31 ^A^	15.25 ± 1.52 ^B^	61.61 ± 1.16 ^A^
PA	0.13 ± 0.01 ^A^	0.170 ± 0.022 ^B^	21.56 ± 1.22 ^A^	18.88 ± 0.89 ^A^	41.70 ± 0.56 ^B^

All values were expressed as mean ± standard error (*n* = 15 for thickness; *n* = 6 for density and MC; *n* = 6 for SW; *n* = 20 for WCA). Means in the same column followed by different capital letters are significantly different (*p* < 0.01). PC control film, PA colorimetric indicator film with high kurugua powder peels, PB colorimetric indicator film with low kurugua powder peels.

**Table 3:** Color parameters (L*, a*, b*, and ΔE*) and opacity (*Op*) of films.

Films	L*	a*	b*	ΔE*	Op (A_600/mm_)
PC	85.59 ± 0.07 ^A^	−20.41 ± 0.05 ^C^	5.22 ± 0.14 ^C^	00.00 ± 0.00 ^C^	2.73 ± 0.62 ^C^
PB	62.31 ± 0.57 ^B^	7.41 ± 0.43 ^B^	9.99 ± 0.29 ^B^	25.02 ± 0.09 ^B^	6.17 ± 0.88 ^B^
PA	51.15 ± 2.43 ^C^	10.79 ± 0.71 ^A^	10.53 ± 0.20 ^A^	36.60 ± 2.47 ^A^	7.74 ± 0.61 ^A^

All values were expressed as mean ± standard error (*n* = 6). Means in the same column followed by different capital letters are significantly different (*p* < 0.01). The ΔE* values of films were calculated using Equation (4) and considering the color parameters of PC as a standard. PC control film, PB colorimetric indicator film with low, and PA colorimetric indicator film with high, kurugua powder peels.

**Table 4:** Color parameters of pH-sensitive films based on cassava starch and kurugua peel powder.

Films	pH	L*	a*	b*	ΔE*
PB	2	79.42 ± 1.47 ^A^	4.14 ± 0.23 ^B^	8.90 ± 0.51 ^F,G,H^	4.04 ± 1.38 ^C,D,E^
	4	79.45 ± 1.00 ^A^	2.66 ± 0.22 ^C^	7.90 ± 0.47 ^H,I^	2.74 ± 0.76 ^E^
	6	77.03 ± 1.35 ^A,B,C^	1.58 ± 0.27 ^D^	7.67 ± 0.26 ^I^	0.00 ± 0.00 ^F^
	8	75.38 ± 1.69 ^B,C,D^	−1.74 ± 0.16 ^F^	11.73 ± 0.20 ^D^	5.70 ± 0.41 ^B,C^
	10	77.84 ± 1.13 ^A,B^	−1.65 ± 0.05 ^F^	14.33 ± 0.20 ^B^	7.55 ± 0.46 ^B^
	12	78.00 ± 0.58 ^A,B^	0.82 ± 0.30 ^E^	10.67 ± 0.34 ^E^	3.51 ± 0.73 ^D,E^
PA	2	75.77 ± 1.43 ^A,B,C^	5.23 ± 0.54 ^A^	8.54 ± 0.59 ^G,H,I^	4.71 ± 1.27 ^C,D^
	4	74.77 ± 1.20 ^C,D,E^	4.45 ± 0.31 ^B^	9.22 ± 0.24 ^F,G,I^	3.76 ± 0.65 ^D,E^
	6	72.81 ± 1.50 ^D,E^	1.99 ± 0.22 ^D^	9.60 ± 0.44 ^F,I^	0.00 ± 0.00 ^F^
	8	72.37 ± 1.38 ^E^	−2.08 ± 0.24 ^F^	13.33 ± 0.78 ^B,C^	5.85 ± 1.02 ^B,C^
	10	75.27 ± 0.99 ^B,C,D,E^	−2.17 ± 0.17 ^F^	17.94 ± 0.33 ^A^	9.78 ± 0.64 ^A^
	12	75.22 ± 1.22 ^B,C,D,E^	0.79 ± 0.20 ^E^	13.11 ± 0.51 ^C^	4.60 ± 1.27 ^C,D,E^

All values were expressed as mean ± standard error (*n* = 6). Means in the same column followed by different capital letters (A, B, C, D, E, F, G, H, I) are significantly different (*p* < 0.01). The ΔE* values of films were calculated using Equation (4) and considering the colorimetric profile as a reference. PB colorimetric indicator film with low kurugua powder peels, PA colorimetric indicator film with high kurugua powder peels.

**Table 5:** Color parameters of cassava starch-based films with kurugua peel powder as a function of the time and storage conditions of chicken meat.

T (°C)	Time (h)	L*	a*	b*	∆E*
4	0	45.44 ± 2.13 ^D^	10.31 ± 0.80 ^A^	8.51 ± 0.51 ^B^	0.00 ± 0.00 ^D^
	24	54.53 ± 1.54 ^C^	7.35 ± 0.62 ^B^	10.78 ± 0.53 ^B^	9.87 ± 3.14 ^C^
	48	63.39 ± 2.30 ^A^	4.48 ± 1.70 ^C^	8.74 ± 3.17 ^B^	19.16 ± 3.15 ^A^
	72	58.47 ± 0.27 ^B^	6.47 ± 0.24 ^B^	10.69 ± 0.33 ^B^	13.77 ± 2.05 ^B,C^
	96	58.58 ±1.13 ^B^	5.88 ± 0.35 ^B,C^	11.39 ± 0.53 ^B^	14.17 ± 2.61 ^A,B,C^
30	0	45.44 ± 2.13 ^D^	10.31 ± 0.80 ^A^	8.51 ± 0.51 ^B^	0.00 ± 0.00 ^D^
	24	56.04 ± 0.94 ^B,C^	2.43 ± 0.43 ^D^	14.96 ± 0.85 ^A^	14.74 ± 2.60 ^A,B,C^
	48	55.82 ± 1.87 ^B,C^	1.28 ± 0.63 ^D^	17.31 ± 2.47 ^A^	16.43 ± 2.92 ^A,B^

All values were expressed as mean ± standard error (*n* = 6). Means in the same column followed by different capital letters are significantly different (*p* < 0.01). The ΔE* values of the films were calculated considering the colorimetric profile at 0 h as a reference.

## Data Availability

The original contributions presented in this study are included in the article. Further inquiries can be directed to the corresponding authors.
